# Supplementary material for: Converting organosulfur compounds to inorganic polysulfides against resistant bacterial infections
Source: Nat Commun. 2018 Sep 13;9:3713. doi: 10.1038/s41467-018-06164-7 (PMC6137151; doi:10.1038/s41467-018-06164-7)
Supplement: Supplementary file 1 — Supplementary Information [file 41467_2018_6164_MOESM1_ESM.pdf]

Supplementary Information

Converting organosulfur compounds to inorganic polysulfides against resistant bacterial infections

Xu *et al.*

Supplementary Figures

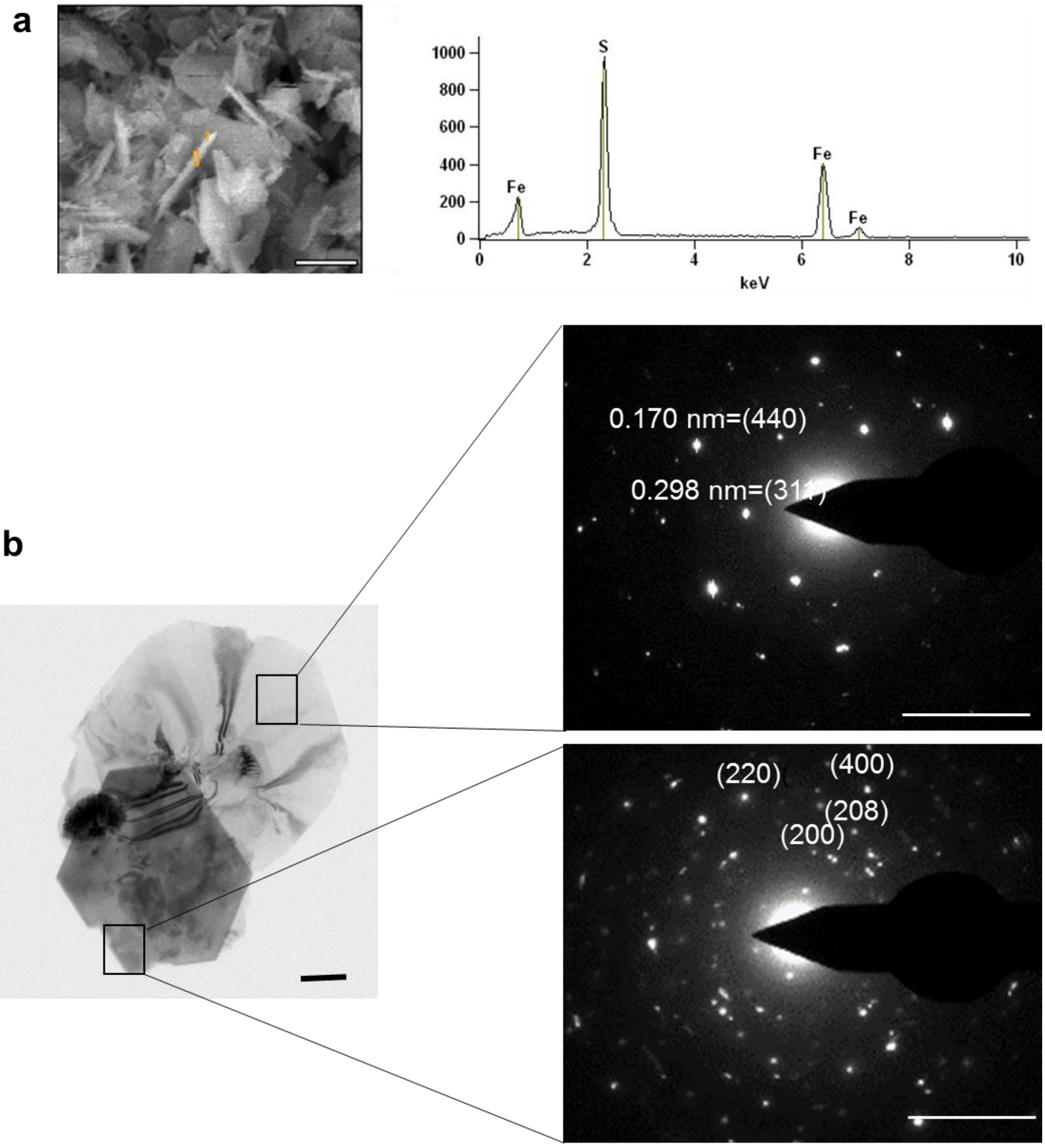

Supplementary Figure 1. Characterizations of synthesized nFeS. a EDS analysis of synthesized nFeS. Scale bar: 5 µm. **b** Selected-area electron diffraction (SAED) pattern of synthesized nFeS. Left scale bar: 200 nm. Right scale bars: 5.00 (1/nm). Representative images are shown.

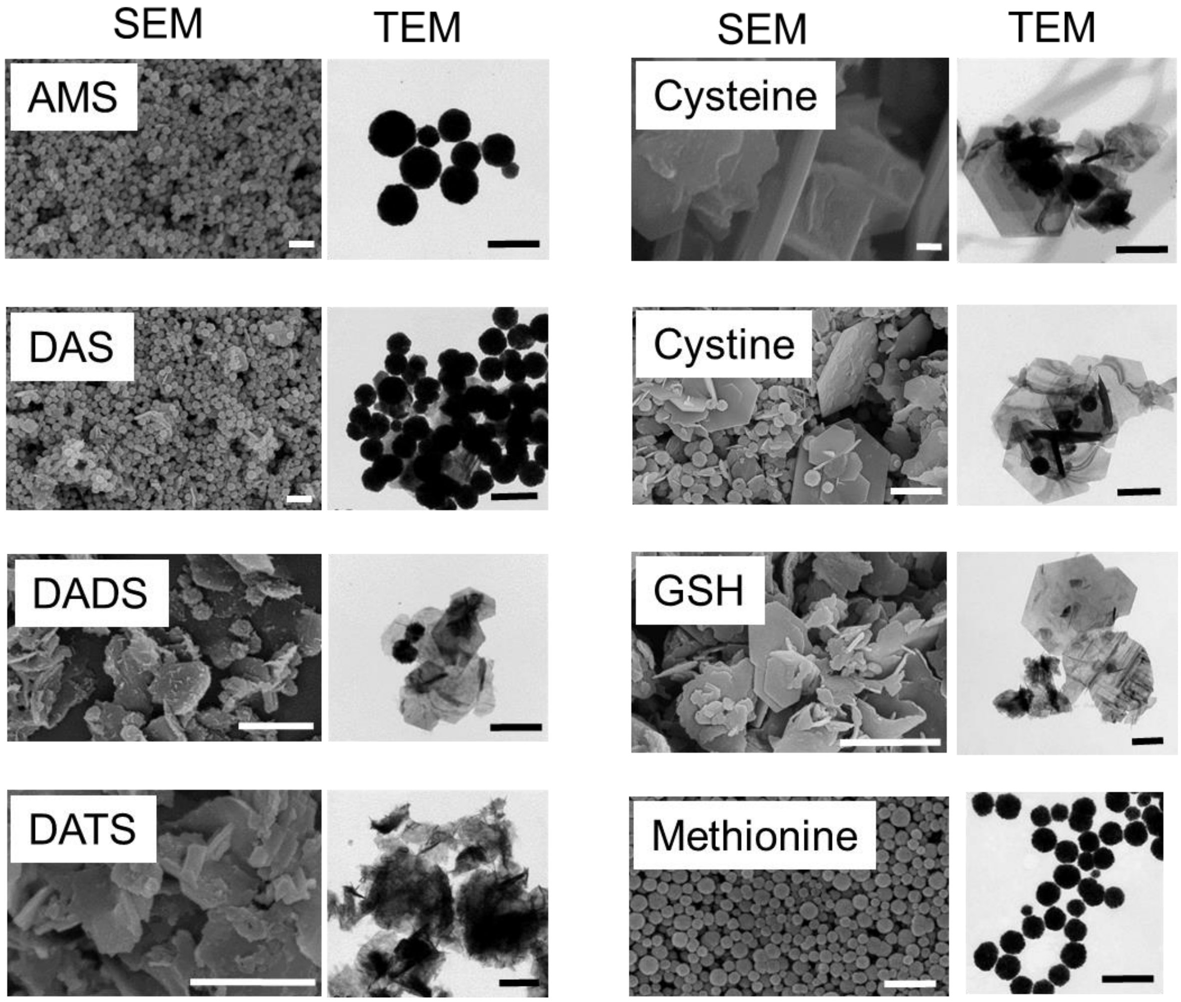

Supplementary Figure 2. SEM and TEM images of converted products from organosulfur compounds. Scale bars in SEM images: 1 μm. Scale bars in TEM images: 0.5 μm. Representative images are shown.

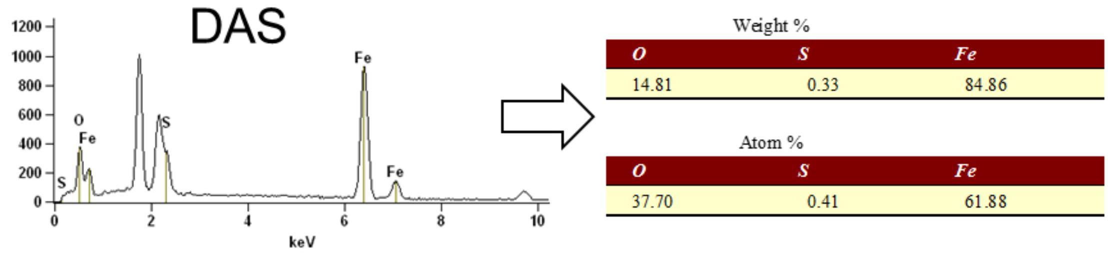

Weight %

O	S	Fe
14.81	0.33	84.86

Atom %

O	S	Fe
37.70	0.41	61.88

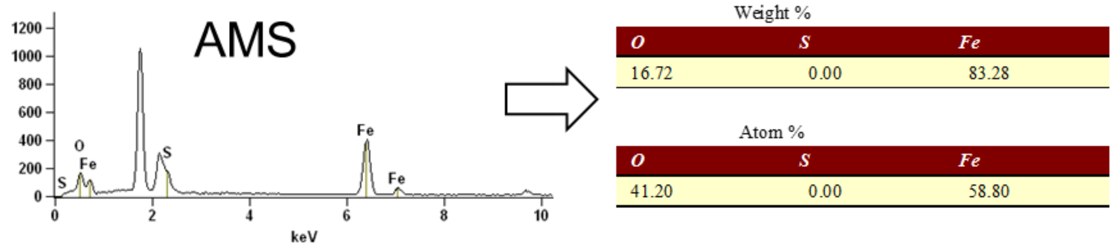

Weight %

O	S	Fe
16.72	0.00	83.28

Atom %

O	S	Fe
41.20	0.00	58.80

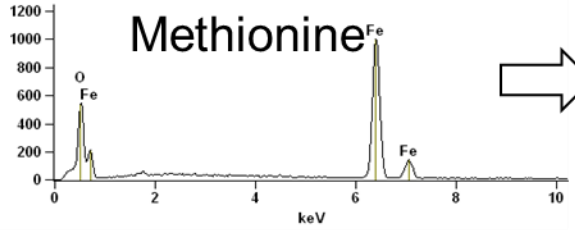

Weight %

O	Fe
20.33	79.67

Atom %

O	Fe
47.11	52.89

Supplementary Figure 3. EDS analysis of the ratio of iron (Fe), sulfur (S) and oxygen (O) in the product from DAS, AMS and Methionine.

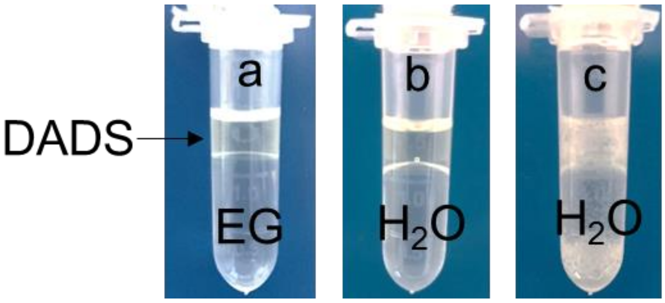

Supplementary Figure 4. Solubility of DADS in ethylene glycol (EG) and water. (a) DADS in EG solvent. (b) DADS in water. (c) DADS in water after shaking.

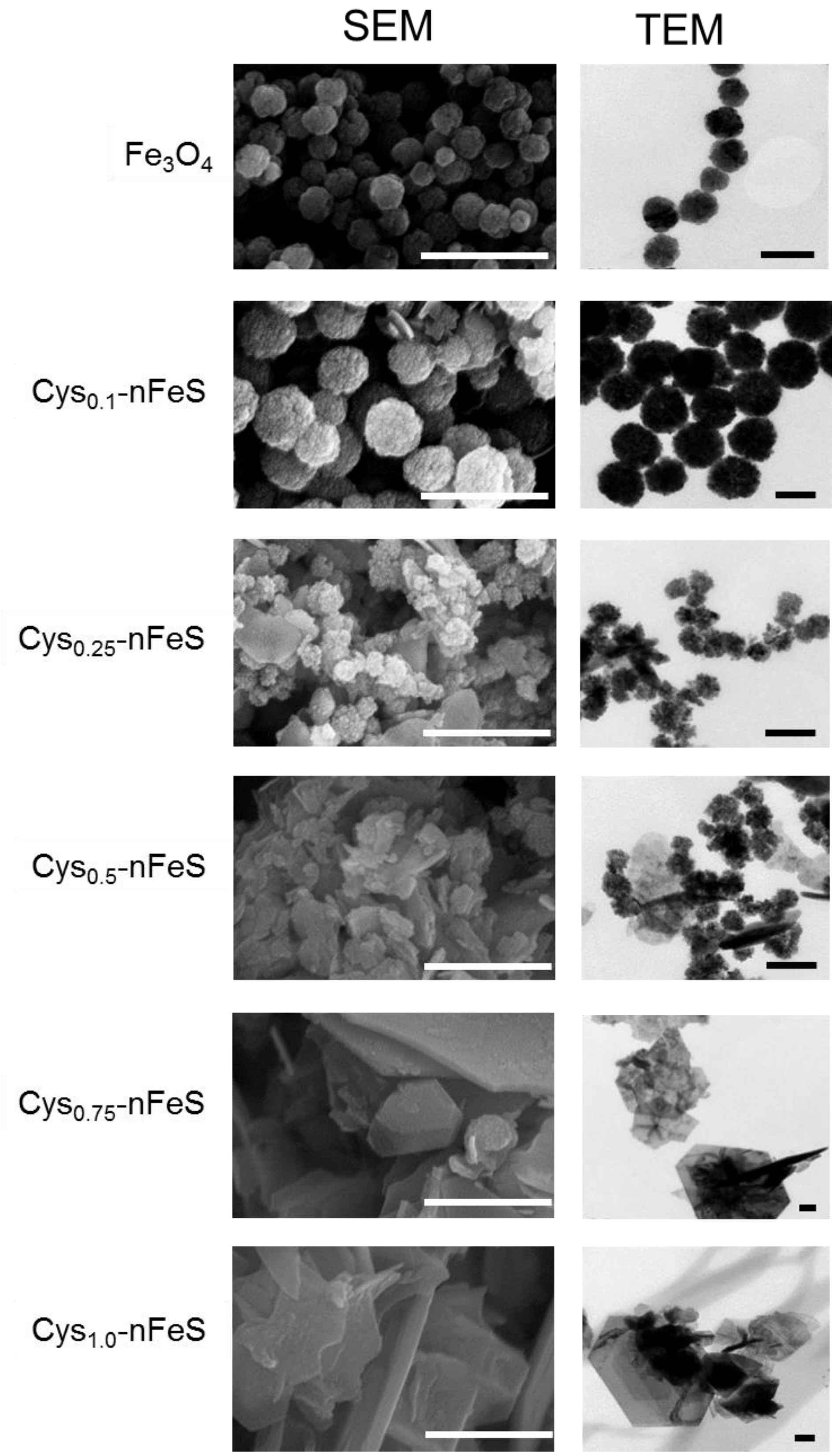

Supplementary Figure 5. SEM and TEM images of Cys-nFeS from different amount of cysteine. Fe_3O_4 represents no cysteine in the reaction. Scale bars in SEM images: 5 μm. Scale bars in TEM images: 200 nm. Representative images are shown.

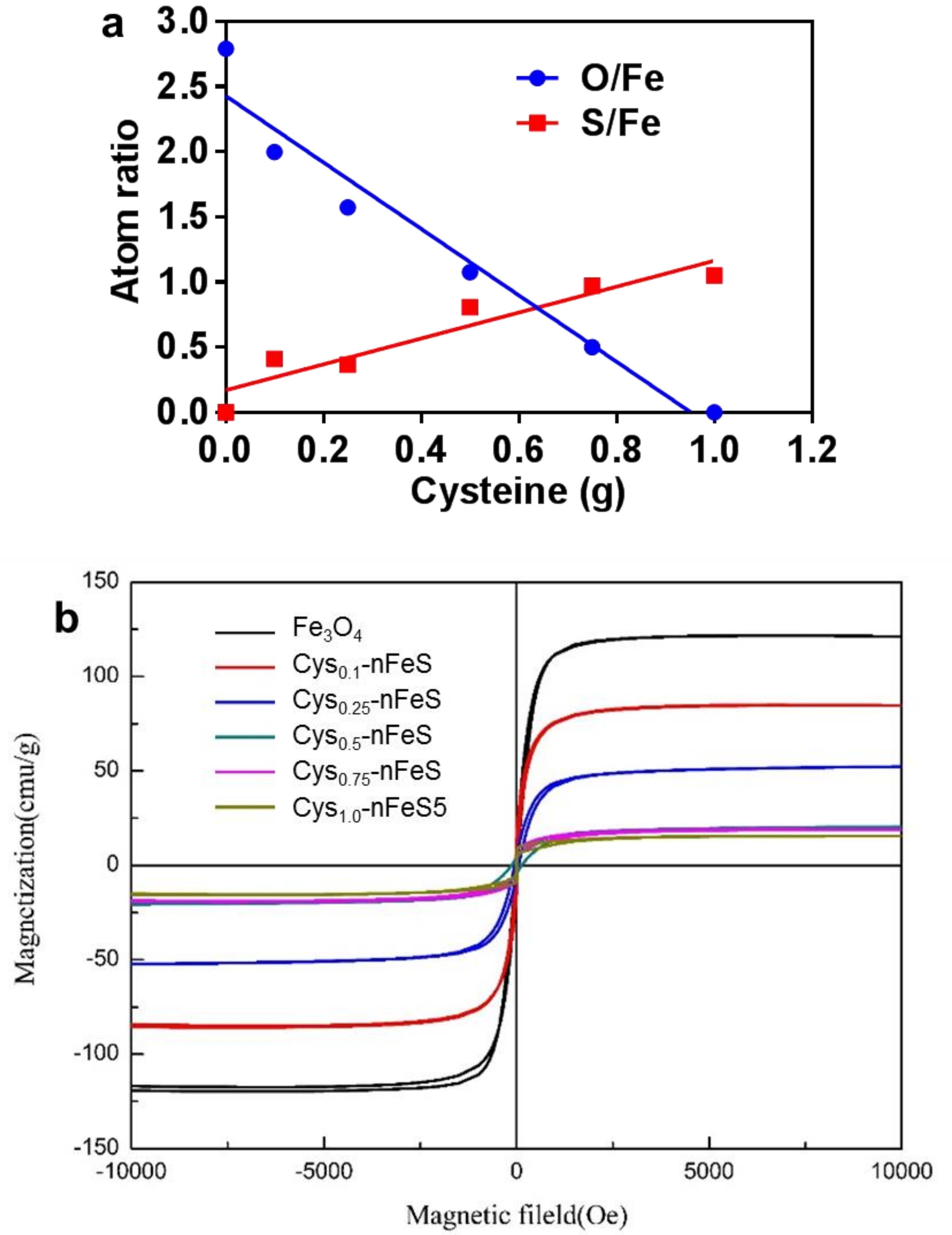

Supplementary Figure 6. Component and magnetization analysis of Cys-nFeS. **a** The change of atom ratio of sulfide to iron and oxygen to iron in the as prepared samples with varied amount of inputting cysteine. **b** The change of magnetization for Cys-nFeS derived from different amount of cysteine.

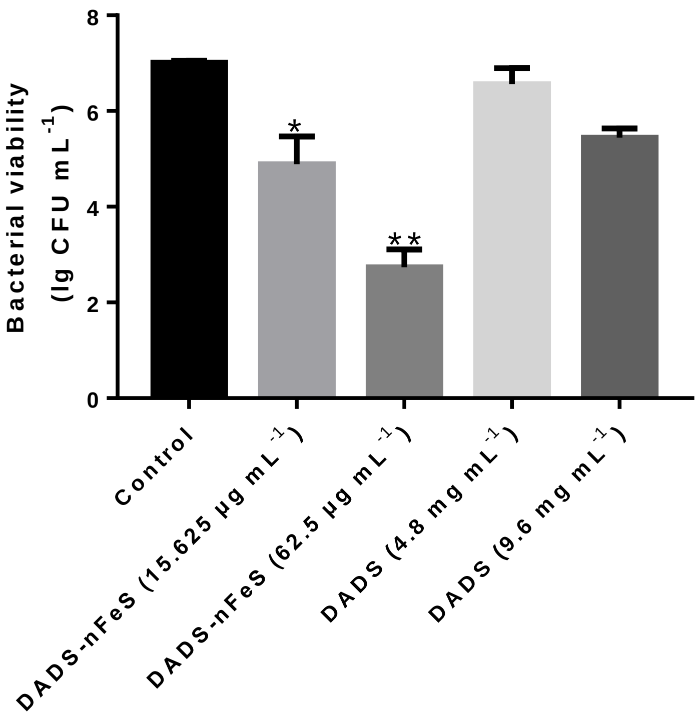

Supplementary Figure 7. Comparison of antibacterial effect on *S. mutans* using DADS and DADS-nFeS. The amount of sulfur in DADS at 9.6 mg/mL was 512-fold higher than that in DADS-nFeS at 15.625 μg/mL. Data are shown as the mean ± s.d. Statistical significance is assessed by unpaired Student's two-sided t-test compared to the control group. $^*p < 0.05$ and $^{**}p < 0.01$. Mean values and error bars were defined as mean and s.d., respectively. All experiments were performed in triplicate.

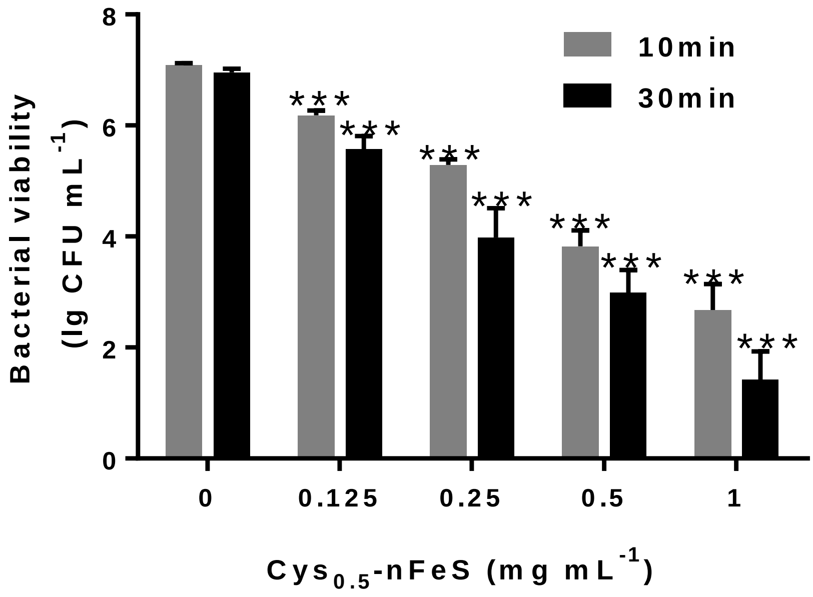

Supplementary Figure 8. Dosage and time dependence of antibacterial activity of Cys$_{0.5}$-nFeS (Tested with *S. mutans* UA159). Error bars shown represent the standard deviation. Data are shown as the mean ± s.d. Statistical significance is assessed by unpaired Student's two-sided t-test compared to the control group. ***$p < 0.001$. Mean values and error bars were defined as mean and s.d., respectively. All experiments were performed in triplicate.

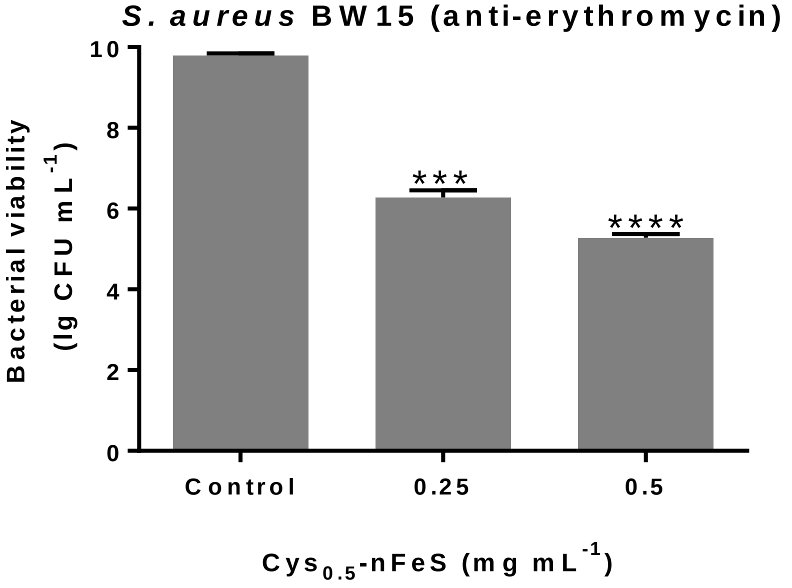

Supplementary Figure 9. Antibacterial activity of Cys0.5-nFeS on erythromycin-resistant *S. aureus* strains. Data are shown as the mean ± s.d. Statistical significance is assessed by unpaired Student's two-sided t-test compared to the control group. Mean values and error bars were defined as mean and s.d., respectively. ***$p < 0.001$ and ****$p < 0.0001$. All experiments were performed in triplicate.

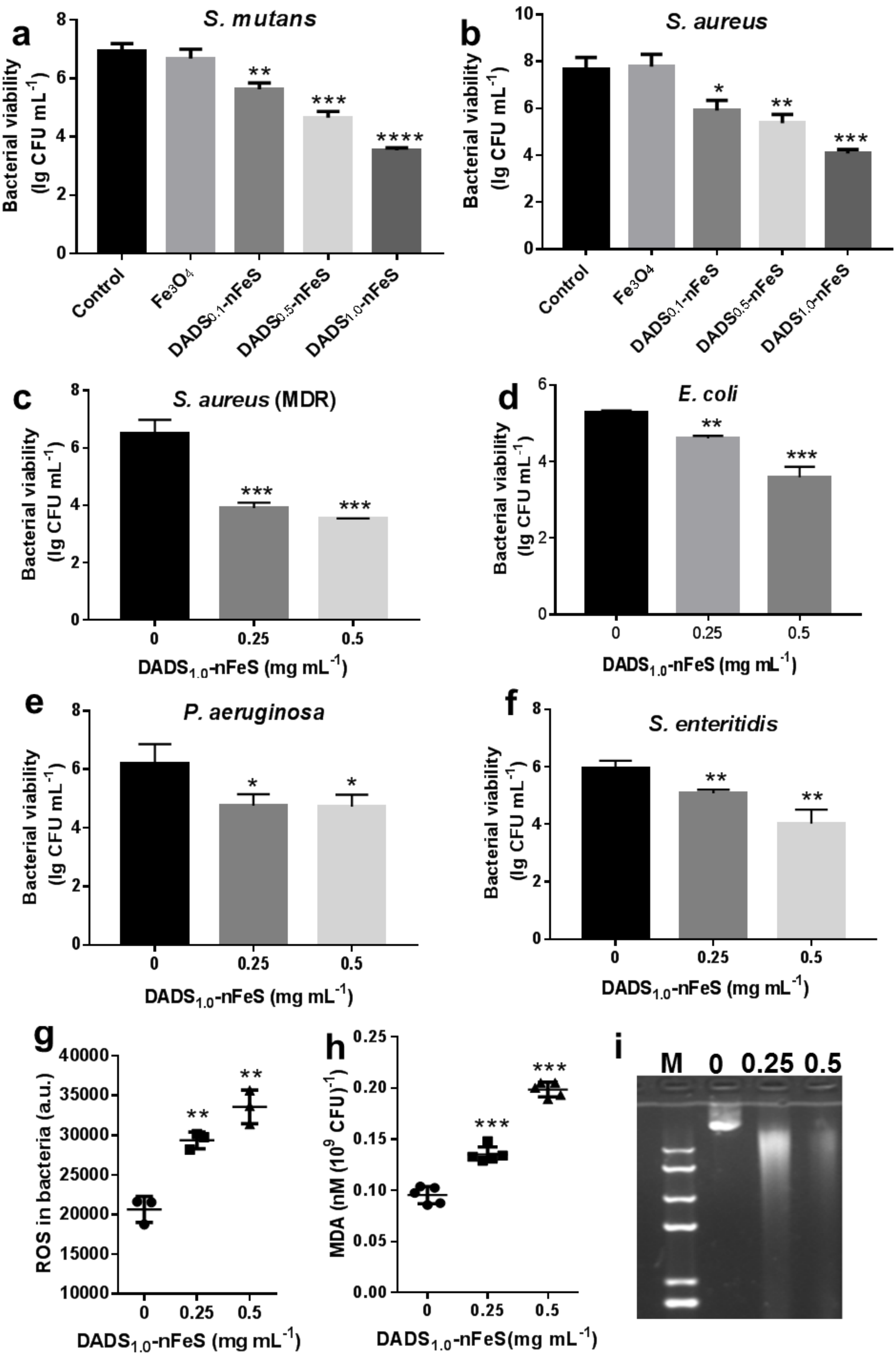

Supplementary Figure 10. Antibacterial activity of DADS-nFeS. a and **b** Antibacterial activity to *S. mutans* and *S. aureus* by DADS-nFeS derived from different

addition of DADS. DADS-nFeS and Fe_3O_4 nanoparticles at 0.5 mg/mL. **c-f** Antibacterial activity of $DADS_{1.0}$-nFeS to *S. aureus* (MDR), *E.coli*, *P. aeruginosa* and *S. enteritidis*. **g** and **h** ROS level and lipid peroxidation of bacteria treated by $DADS_{1.0}$-nFeS. **i** Genomic DNA degradation of bacteria treated by $DADS_{1.0}$-nFeS. M: DNA marker. Data are shown as the mean ± s.d. Statistical significance is assessed by unpaired Student's two-sided t-test compared to the control group. **p < 0.01, ***p < 0.001 and ****p < 0.0001. Mean values and error bars were defined as mean and s.d., respectively. All experiments were performed in triplicate, and representative images are shown.

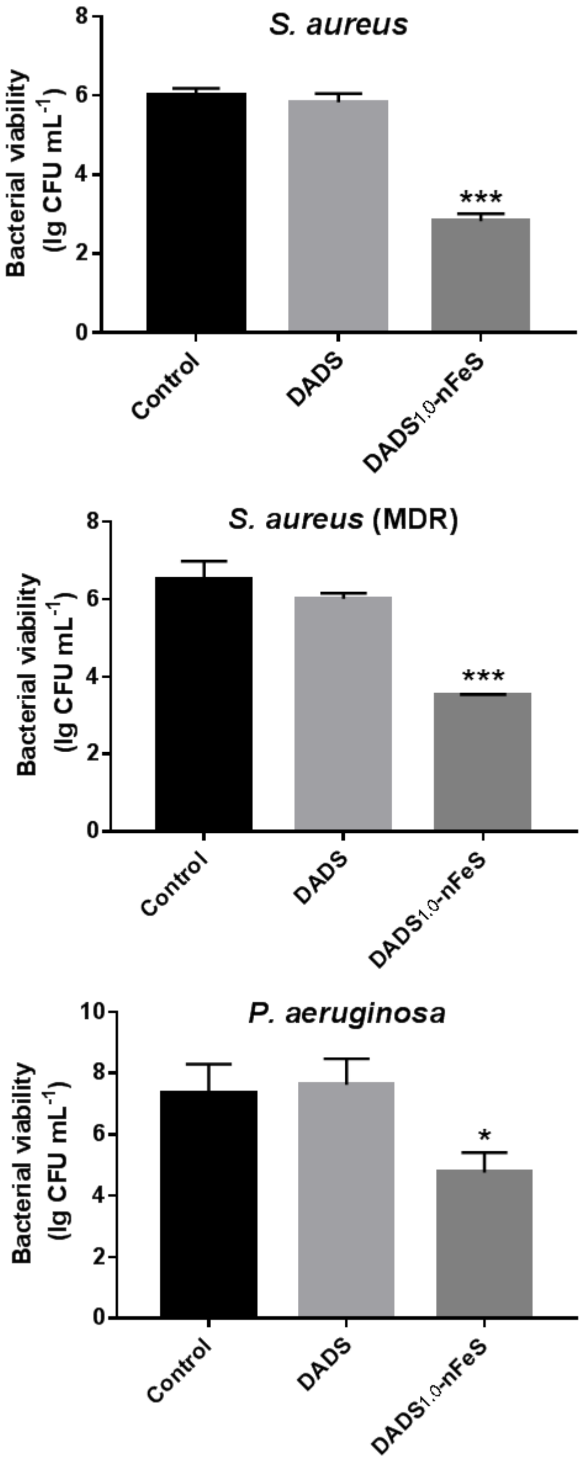

Supplementary Figure 11. Antibacterial activity of DADS and DADS-nFeS to *S. aureus*, *S. aureus* (MDR) and *P. aeruginosa*. The DADS concentration was normalized to the same sulfur content in $DADS_{1.0}$-nFeS at 0.5 mg/mL. Data are shown as the mean ± s.d. Statistical significance is assessed by unpaired Student's two-sided t-test compared to the control group. $^{*}p < 0.05$, $^{***}p < 0.001$. Mean values and error bars were defined as mean and s.d., respectively. All experiments were performed in triplicate.

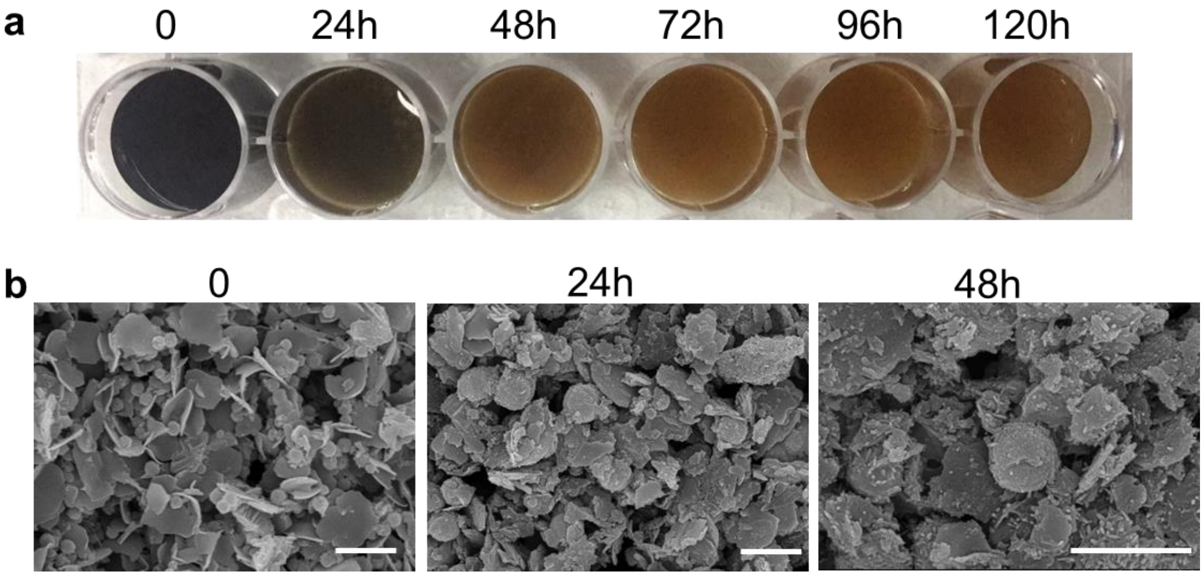

Supplementary Figure 12. Color change and nanostructure transformation of nFeS. a Color change of DADS-nFeS incubated in water at different time. **b** SEM image of nanostructure transformation of DADS-nFeS after incubation in water. Scale bars: 1 µm. All experiments were performed in triplicate, and representative images are shown.

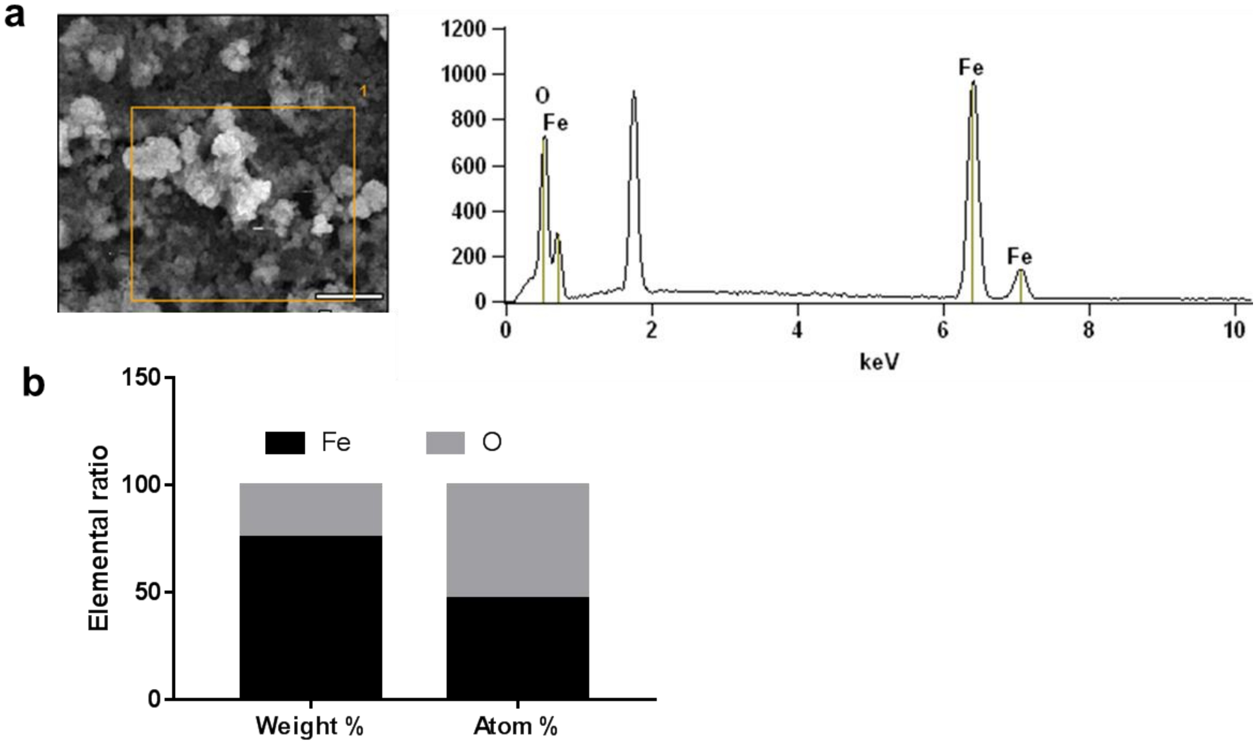

Supplementary Figure13. EDS analysis of the precipitate of Cys-nFeS incubated in water for 96h. **a** EDS analysis of nFeS precipitates. Scale bar: 5 µm. **b** The ratio of iron (Fe) and oxygen (O) in nFeS precipitate. Representative images are shown.

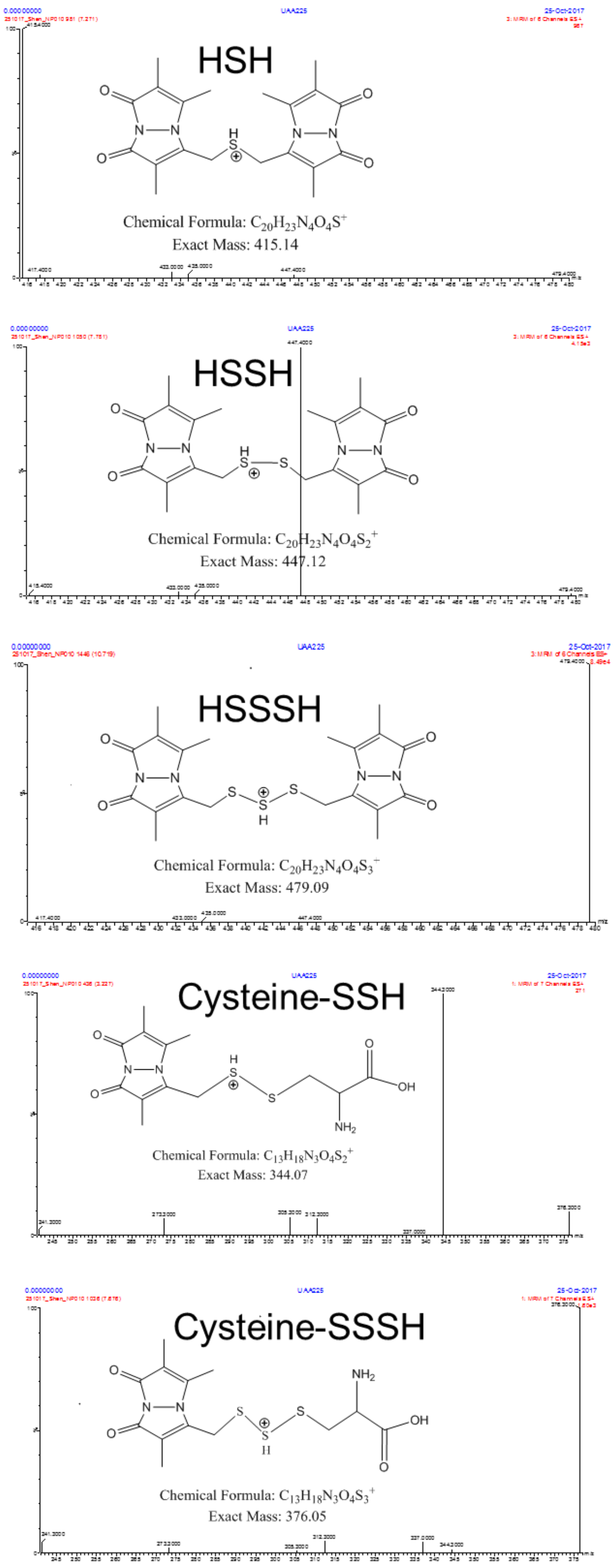

Supplementary Figure 14. HPLC-MS analysis of sulfides in the supernatant of Cys-nFeS dissolved in water for 48 h. (All sulfides reacted with MBB).

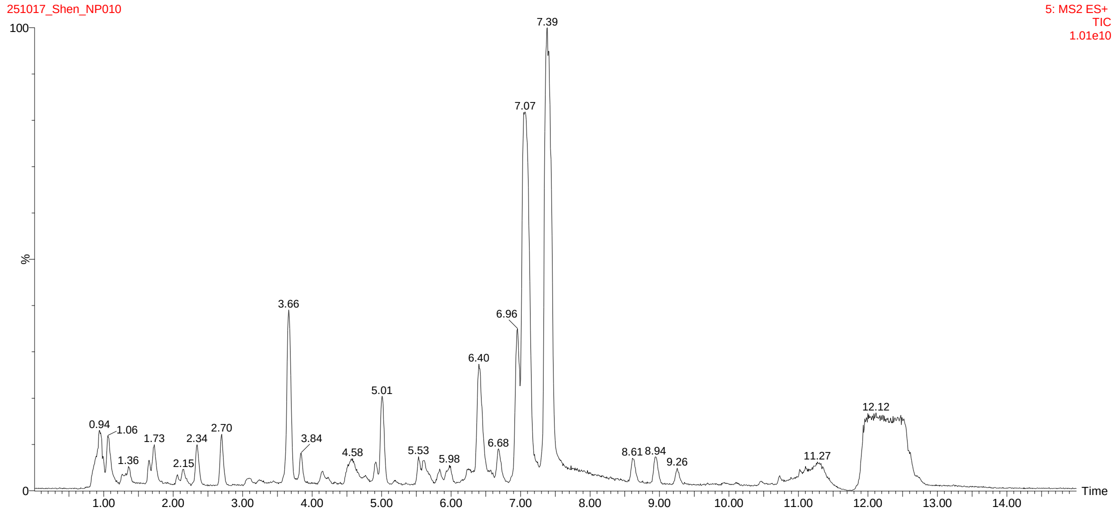

Supplementary Figure 15. Original map of LC-MS analysis for the supernatant of Cys1.0-nFeS dissolved in water for 48h.

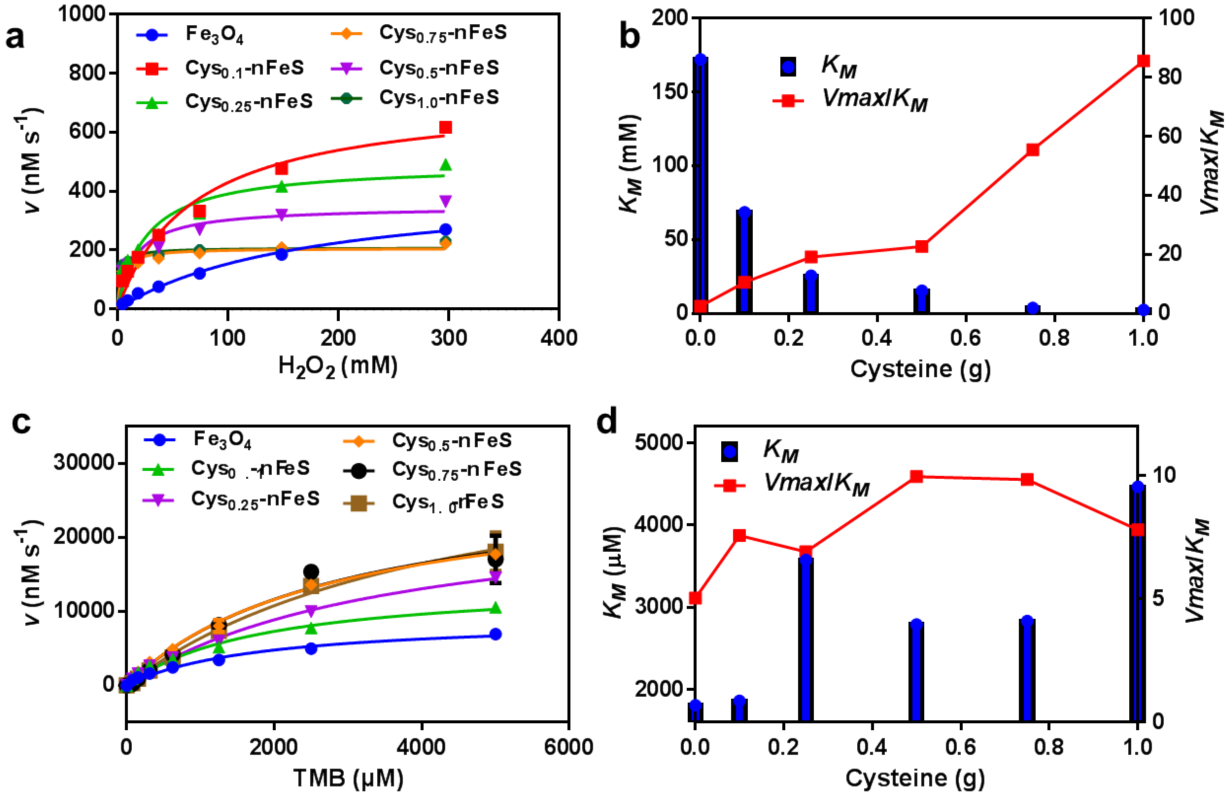

Supplementary Figure 16. **Kinetics assay of peroxidase-like activity of Cys-nFeS.** **a** Michaelis-Menten kinetics of peroxidase-like activity of Cys-nFeS (with varied [H_2O_2]). **b** The trend of K_M and the ratio of $Vmax/K_M$ in the kinetics assay with varied [H_2O_2]. **c** Michaelis-Menten kinetics of peroxidase-like activity of Cys-nFeS (with varied [TMB]). **d** The trend of K_M and the ratio of $Vmax/K_M$ in the kinetics assay with varied [TMB], respectively.

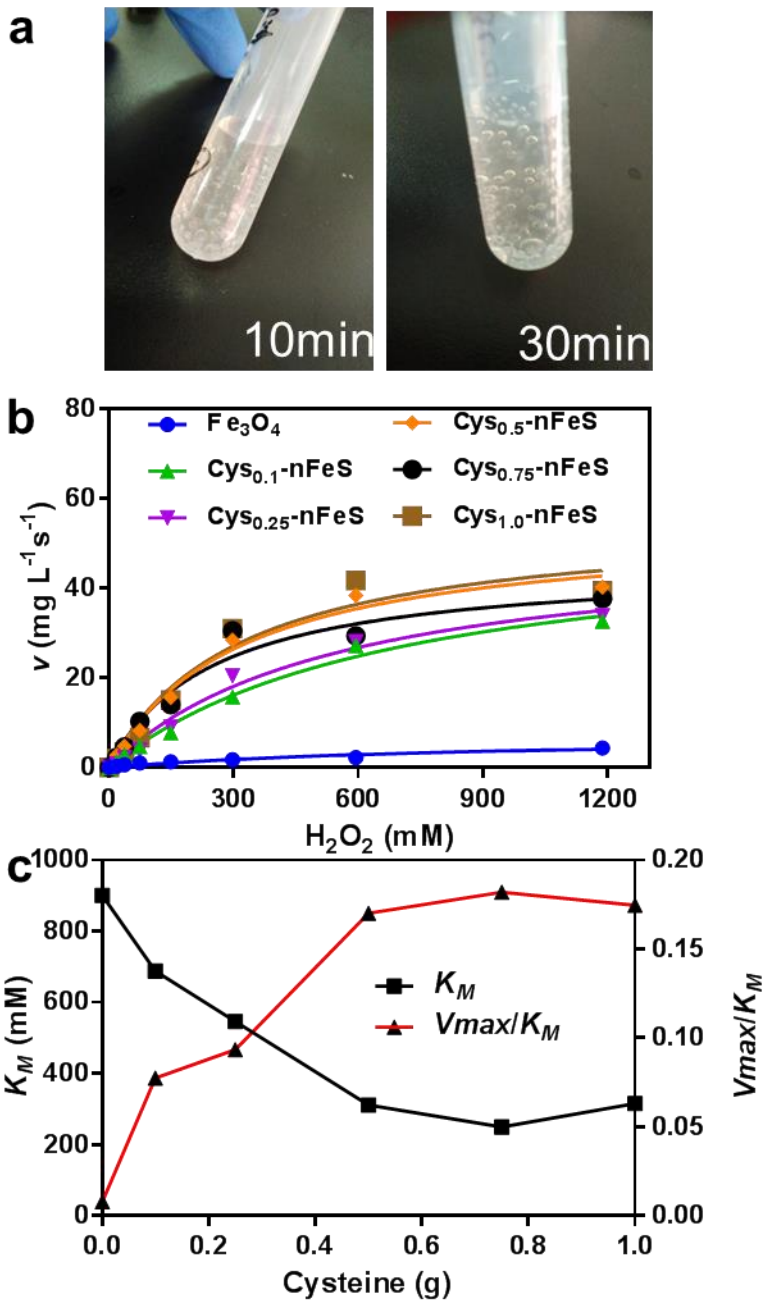

Supplementary Figure 17. Catalase-like activity of Cys-nFeS. **a** The catalase-like activity to decompose H_2O_2 into oxygen (bubble) by Cys-nFeS. Representative images are shown. **b** The trend of K_M and the ratio of *Vmax*/K_M in the kinetics assay with varied cysteine. **c** The trend of K_M and the ratio of *Vmax*/K_M in the kinetics assay with varied [H_2O_2].

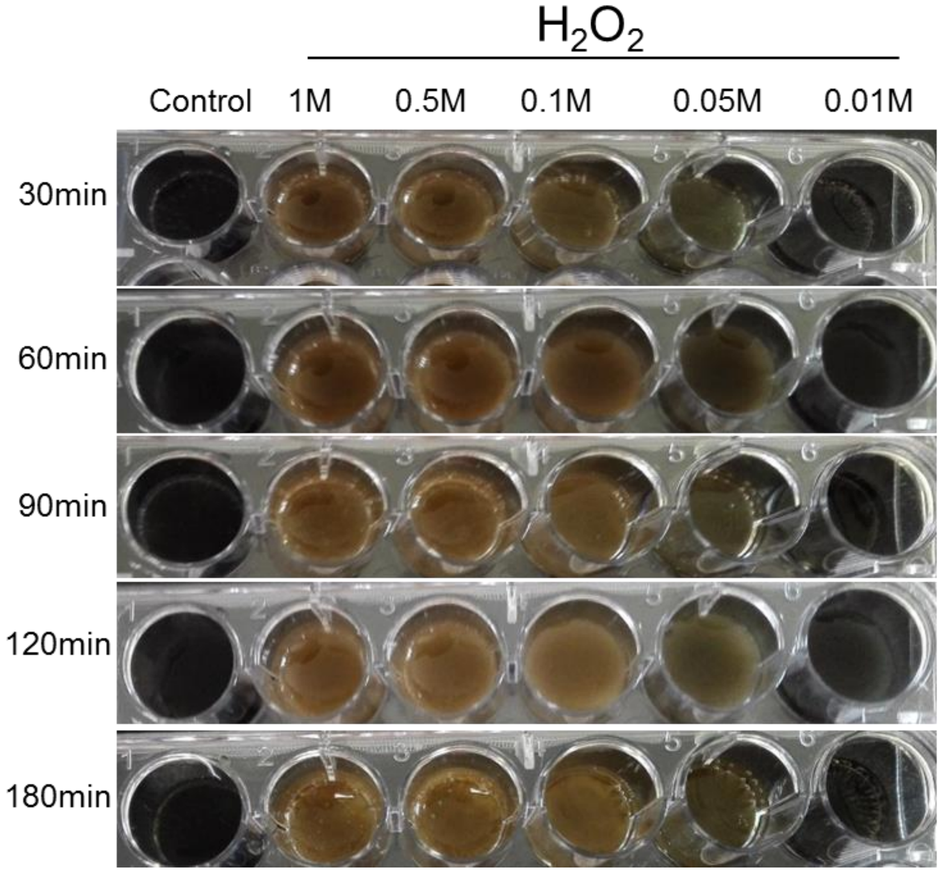

Supplementary Figure 18. H_2O_2 stimulated sulfide release with color change. Representative images are shown.

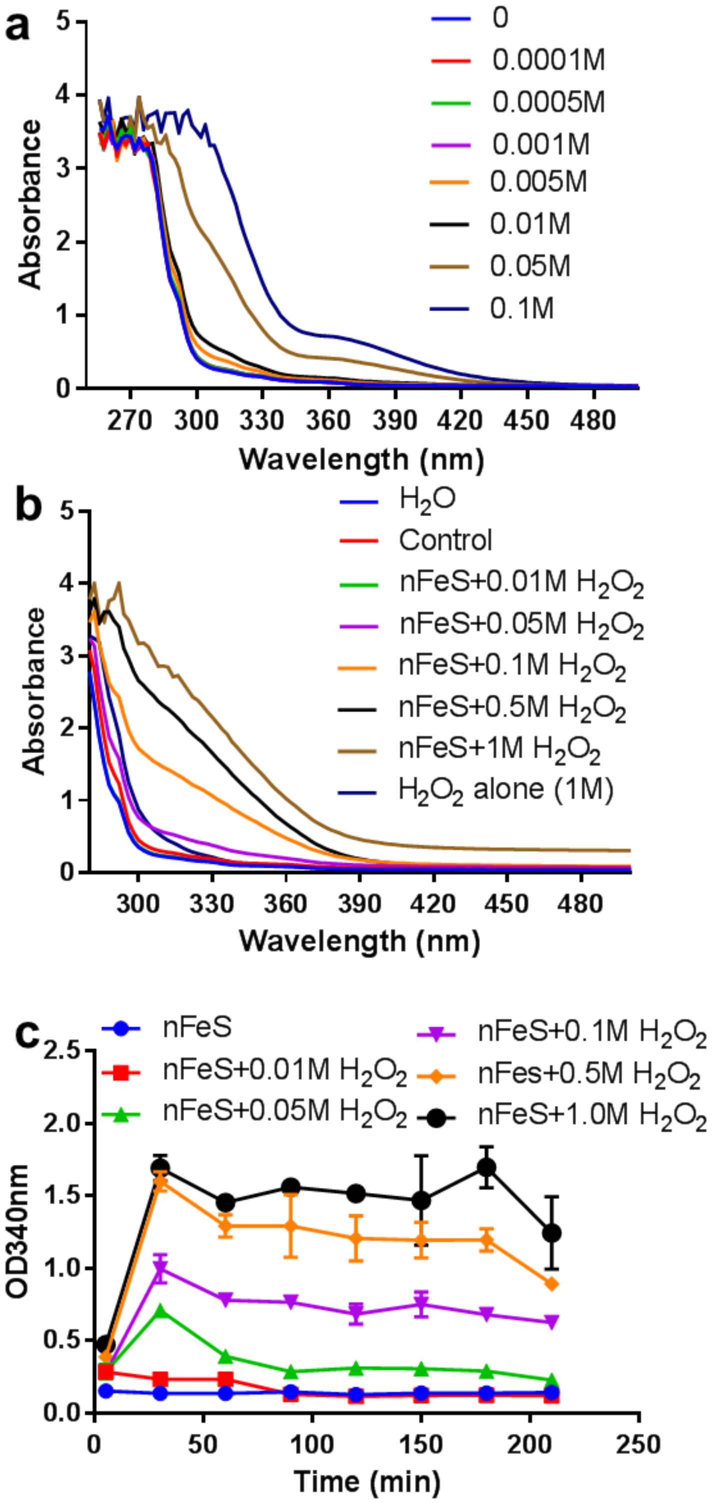

Supplementary Figure 19. Characterization release behavior of polysulfanes stimulated by H_2O_2. **a** Absorbance spectrum of NaHS with varied concentrations in water. **b** Raw absorbance scanning of sulfide release in the supernatant of $Cys_{0.5}$-nFeS solution. **c** Absorbance at 340nm represents sulfide release relevant to the concentration of H_2O_2.

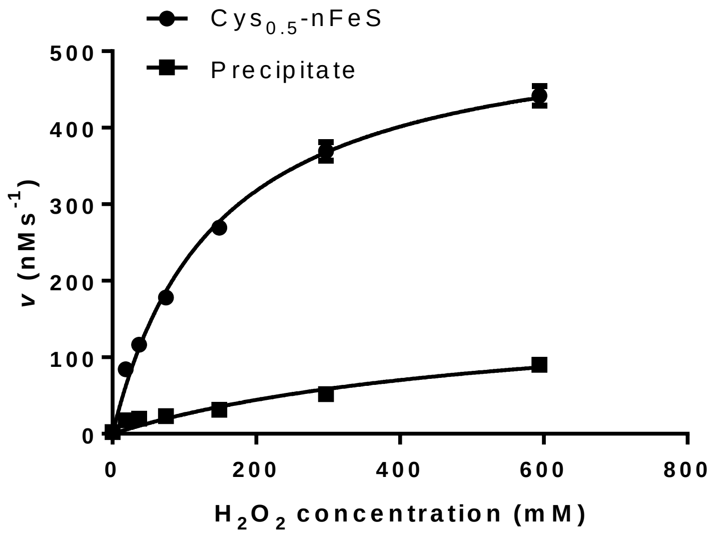

Supplementary Figure 20. Kinetics of peroxidase-like activity of the precipitate and original Cys-nFeS.

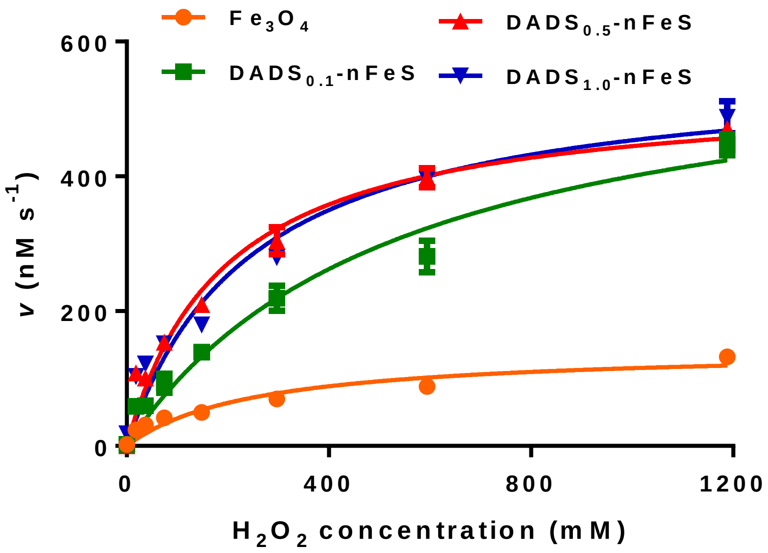

Supplementary Figure 21. **Confirmation of peroxidase-like activity of DADS-nFeS.** The catalysis follows Michaelis-Menten kinetics of (with varied [H_2O_2]).

a

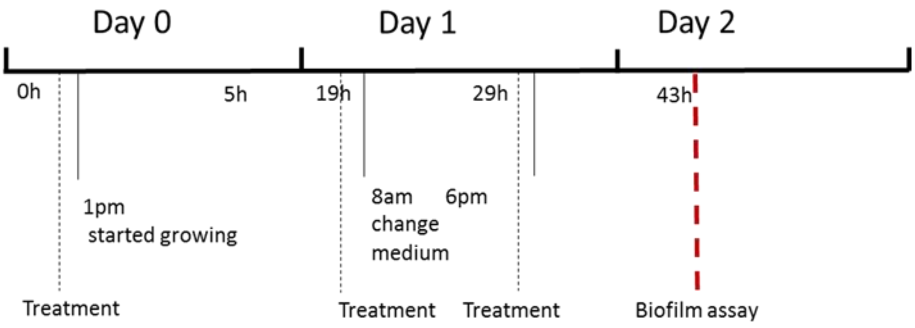

b

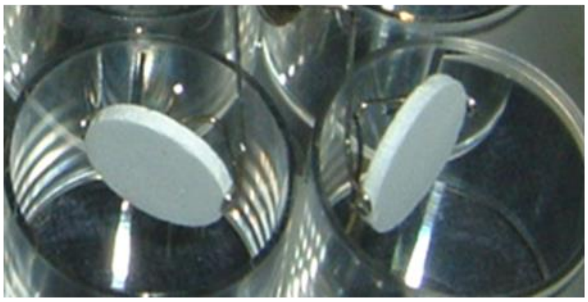

Supplementary Figure 22. Dental biofilm established with *S. mutans* UA159 to mimic clinical situation. **a** The timeline for biofilm culture and treatments. **b** A demo of vertical hydroxyapatite discs (HA) in the culture well for biofilm formation. Reprinted from Biomaterials, 101, Lizeng Gao, Yuan Liu, Dongyeop Kim, Yong Li, Geelsu Hwang, Pratap C. Naha, David P. Cormode, Hyun Koo, Nanocatalysts promote *Streptococcus mutans* biofilm matrix degradation and enhance bacterial killing to suppress dental caries *in vivo*, 272-284, Copyright (2016), with permission from Elsevier. Representative images are shown.

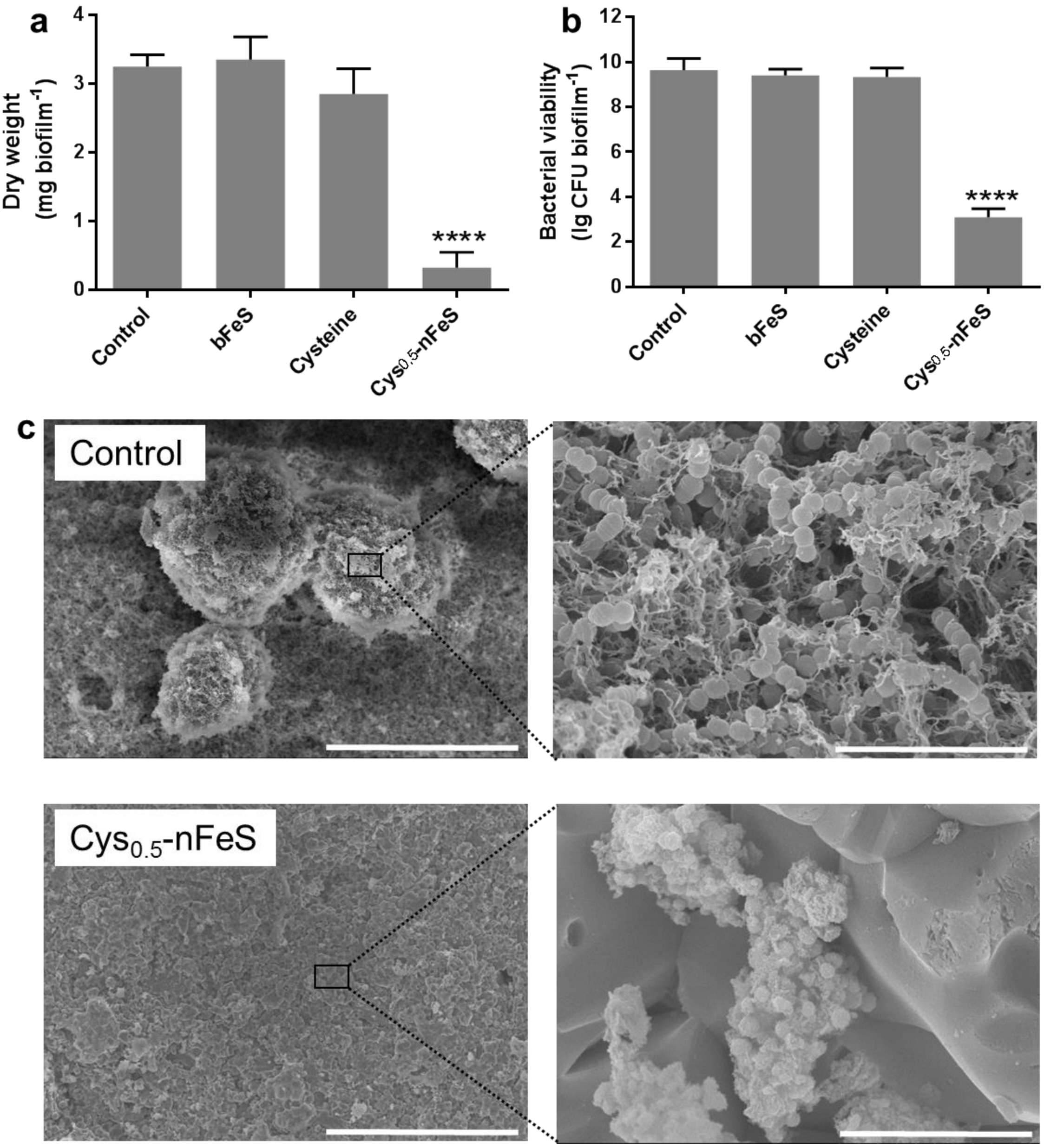

Supplementary Figure 23. Biofilm prevention with Cys-nFeS on saliva-coated HA (sHA). **a** and **b** Biofilm assays for dry weight and cell viability after treatment with Cys-nFeS. Data are shown as the mean ± s.d. Statistical significance is assessed by unpaired Student's two-sided t-test compared to the control group. Mean values and error bars were defined as mean and s.d., respectively. ****$p < 0.0001$. **c** SEM image of dental biofilm treated with Cys-nFeS. Left scale bars: 100 µm. Right scale bars: 5 µm. All experiments were performed in triplicate with representative images shown.

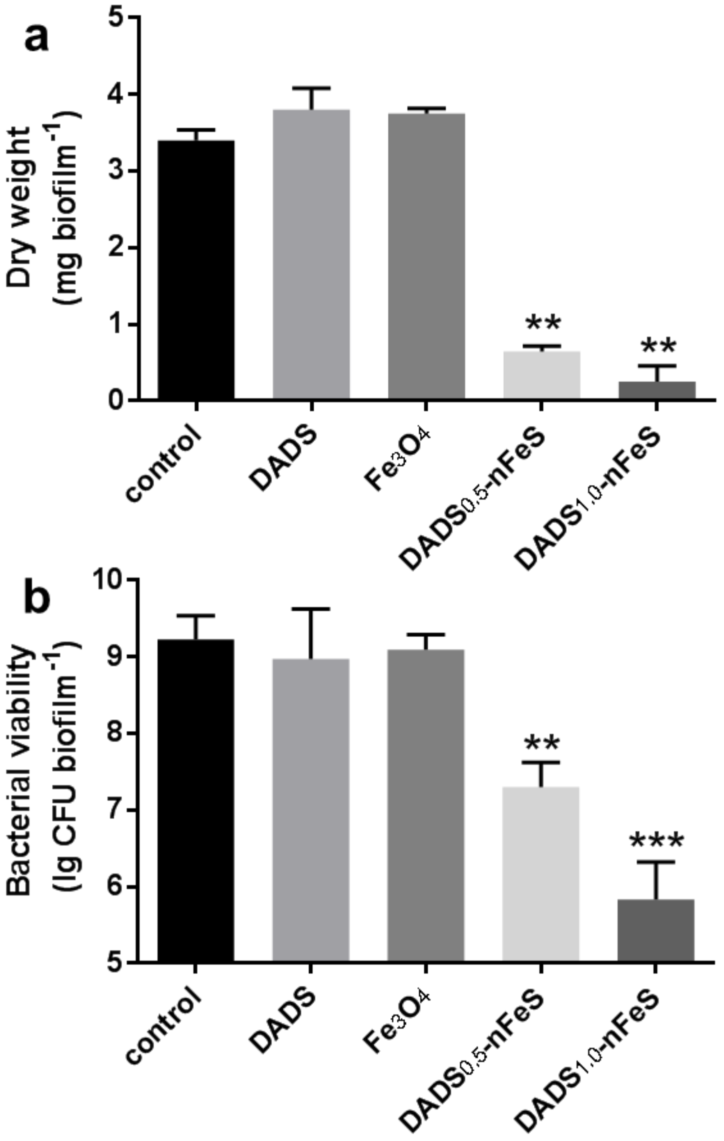

Supplementary Figure 24. Dental Biofilm prevention with DADS-nFeS on saliva-coated HA (sHA). **a** Dry weight of dental biofilm. **b** Bacterial viability in the biofilm. Error bars shown represent the standard deviation. Data are shown as the mean ± s.d. Statistical significance is assessed by unpaired Student's two-sided t-test compared to the control group. $^{**}p < 0.01$, and $^{***}p < 0.001$. Mean values and error bars were defined as mean and s.d., respectively. All experiments were performed in triplicate.

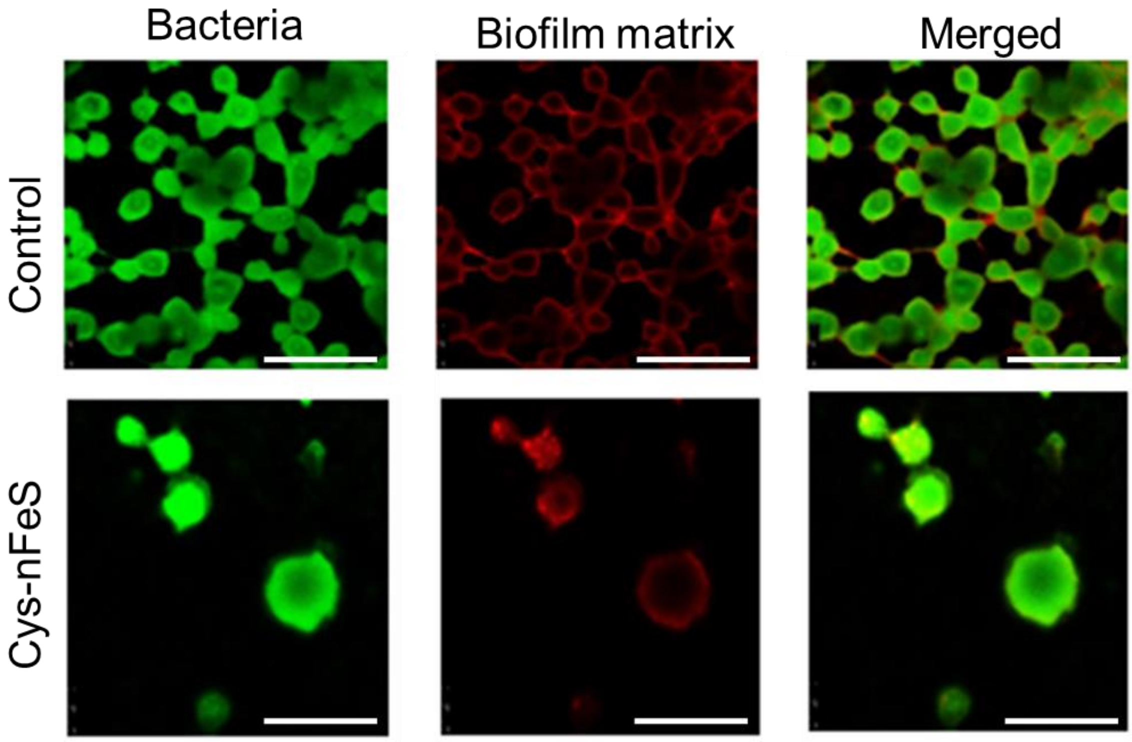

Supplementary Figure 25. Confocal image of dental biofilm treated with Cys-nFeS. Scale bars: 200 μm. All experiments were repeated in triplicate with representative images shown.

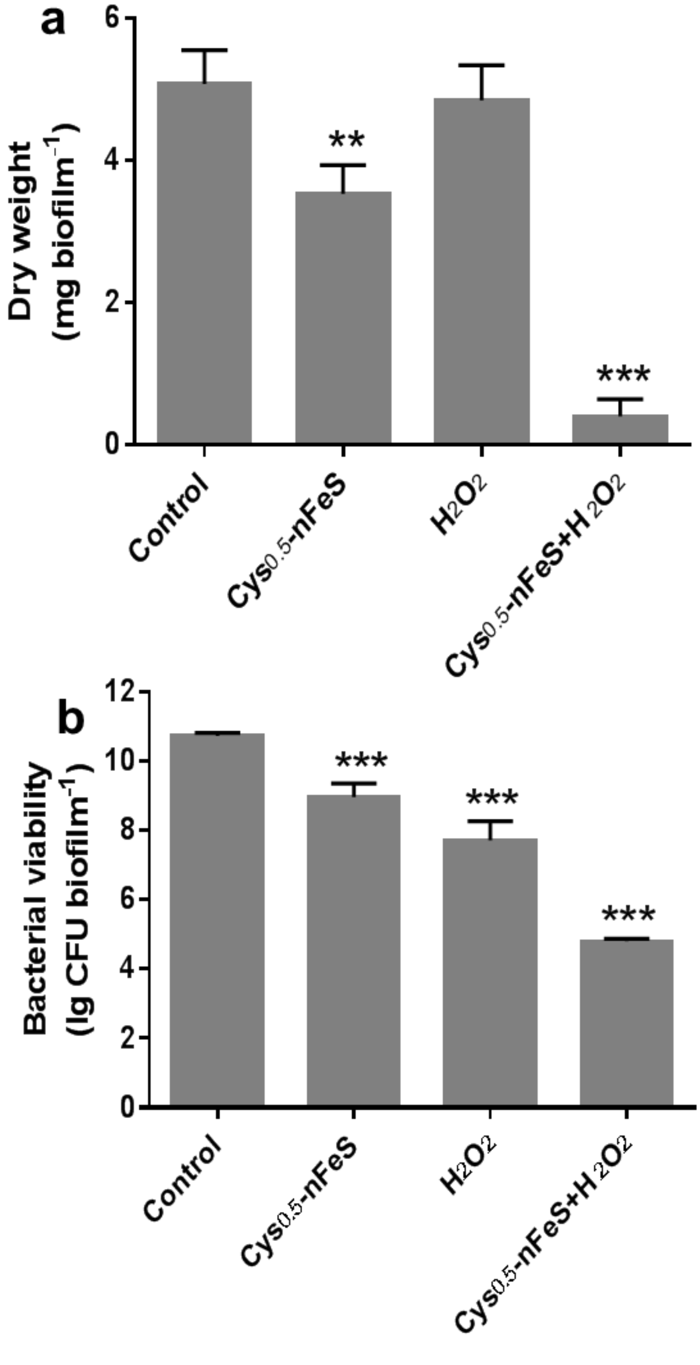

Supplementary Figure 26. Combining Cys-nFe with H_2O_2 to eliminate *S. mutans* biofilm. a Dry weight of dental biofilm. **b** Bacteria viability in the biofilm. $Cys_{0.5}$-nFeS at 0.5 mg/mL and H_2O_2 at 0.5%. Data are shown as the mean ± s.d. Statistical significance is assessed by unpaired Student's two-sided t-test compared to the control group. $^{**}p < 0.01$, and $^{***}p < 0.001$. Mean values and error bars were defined as mean and s.d., respectively. All experiments were performed in triplicate.

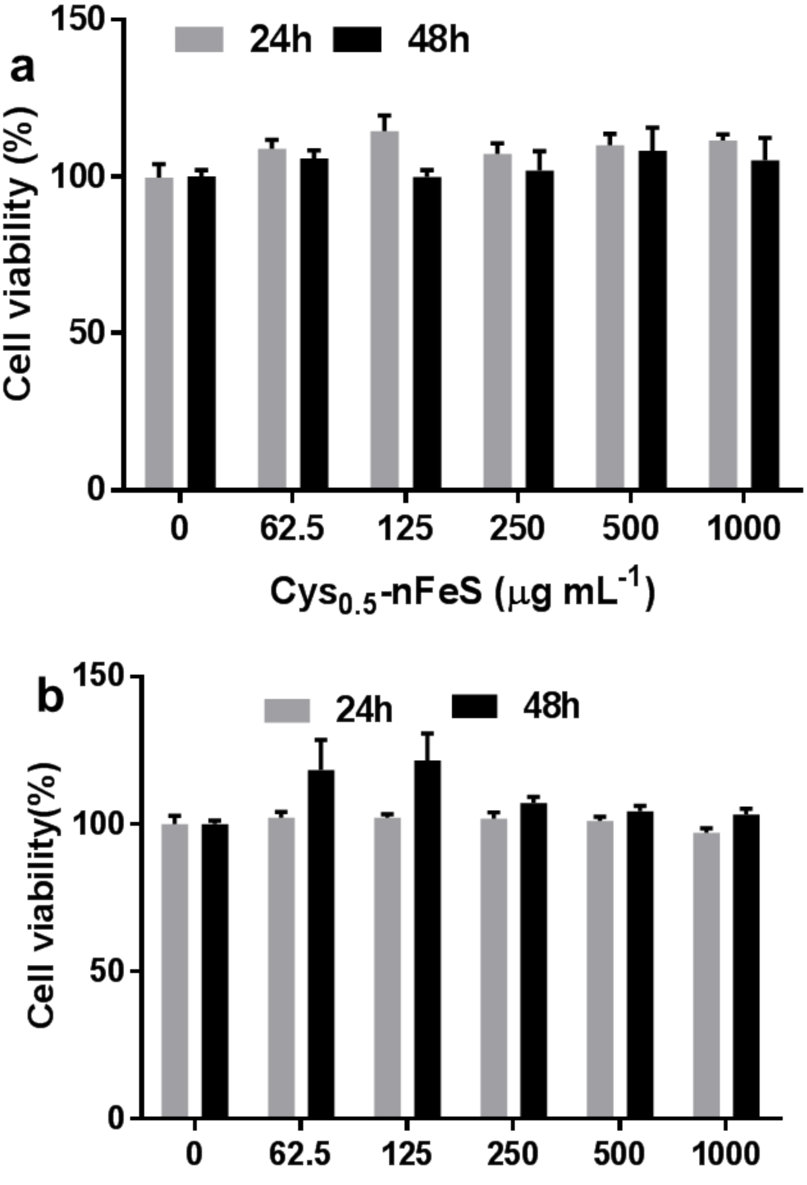

Supplementary Figure 27. Influence of nFeS on HOK cells. a Cell viability of HOK cell treated with Cys-nFeS solution. **b** Cell viability of HOK cell treated with the supernatant from Cys-nFeS solution in (**a**) (96 h incubation). Data are shown as the mean ± s.d. Mean values and error bars were defined as mean and s.d., respectively. All experiments were performed in triplicate.

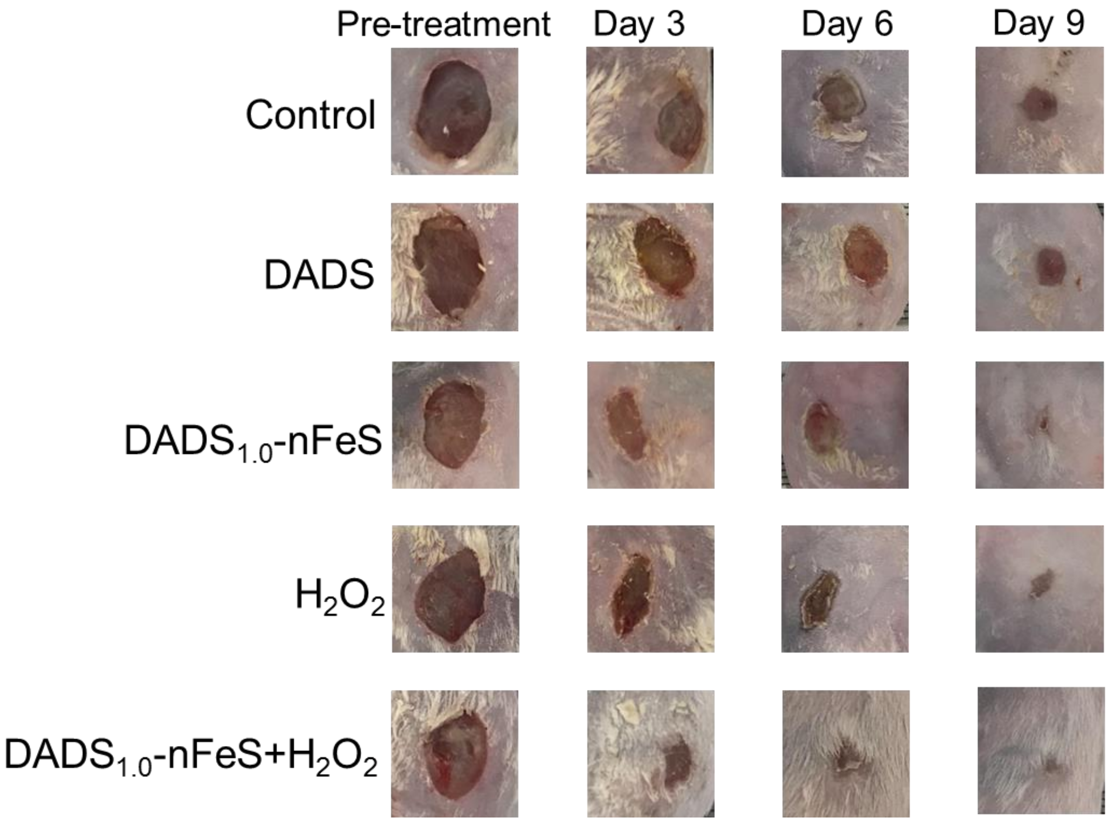

Supplementary Figure 28. Infected-wound healing with DADS-nFeS. Photographs of *P. aeruginosa* infected wound treated with buffer (control), DADS, DADS$_{1.0}$-nFeS, H_2O_2, and DADS$_{1.0}$-nFeS + H_2O_2 at different times. (5 mice in each group). Representative images are shown.

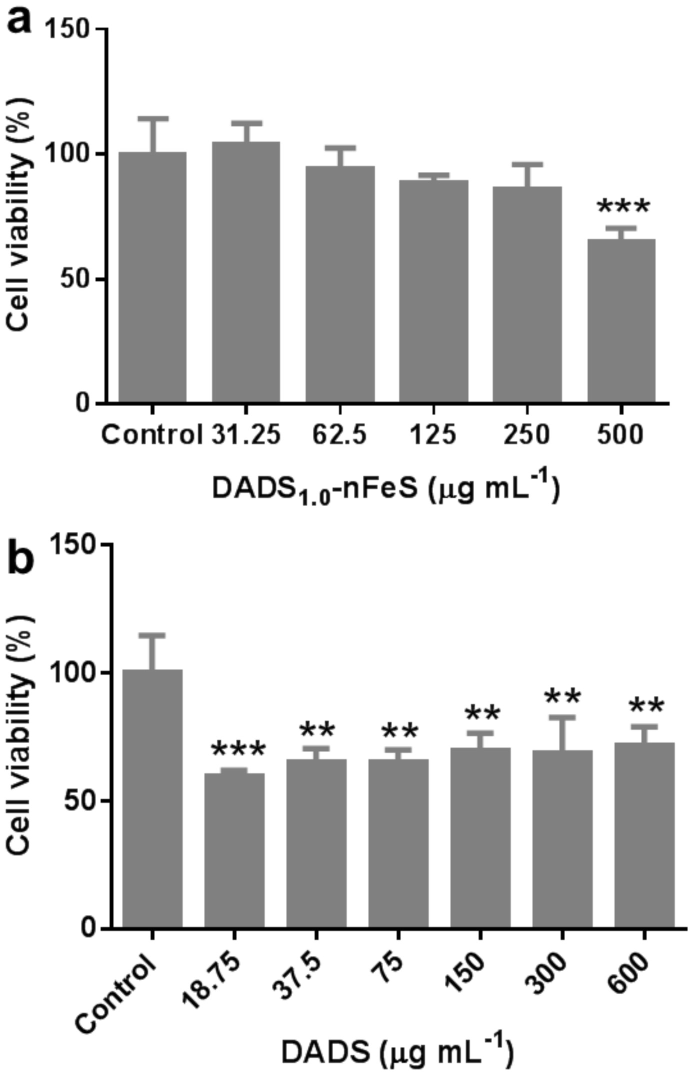

Supplementary Figure 29. Influence of $DADS_{1.0}$-nFeS and DADS on the viability of fibroblast cells. **a** 3T3 cell viability treated with $DADS_{1.0}$-nFeS under different concentrations. **b** 3T3 cell viability treated with DADS under different concentrations. Data are shown as the mean ± s.d. Statistical significance is assessed by unpaired Student's two-sided t-test compared to the control group. **p < 0.01, and ***p < 0.001. Mean values and error bars were defined as mean and s.d., respectively. All experiments were performed in triplicate.

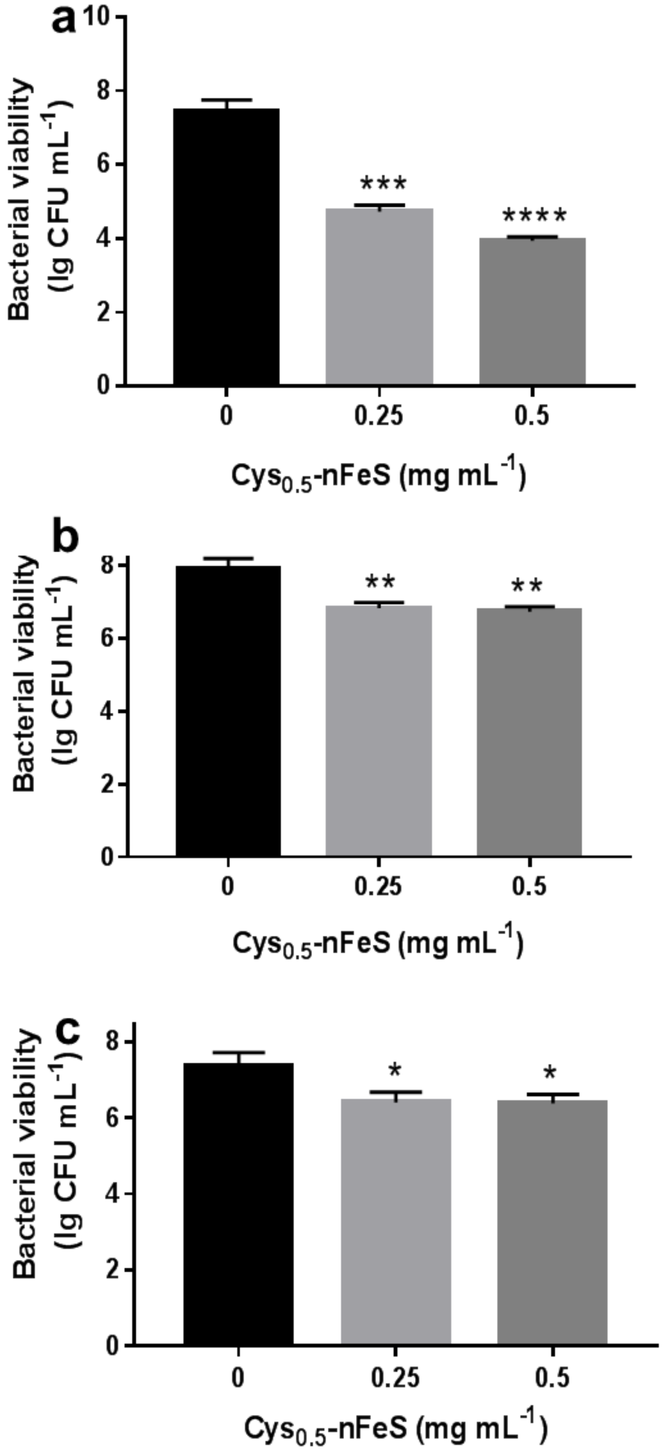

Supplementary Figure 30. Antibacterial activity of cys$_{0.5}$-nFeS to *S. mutans* in different medium. **a** In NaCl. **b** In RPMI-1640 medium containing FBS 10%. **c** In plasma of mouse serum. Data are shown as the mean ± s.d. Statistical significance is assessed by unpaired Student's two-sided t-test compared to the control group. *$p < 0.05$, **$p < 0.01$, ***$p < 0.001$ and ****$p < 0.0001$. Mean values and error bars were defined as mean and s.d., respectively. All experiments were performed in triplicate.

Supplementary Tables

Supplementary Table 1. Atom ratio (%) in Cys-nFeS from different amount of cysteine addition.

Cys (g)	O (Atom %)	Fe (Atom %)	S (Atom %)
0	42.33	15.16	0
0.1	37.57	18.79	7.72
0.25	32.56	20.7	7.57
0.5	30.12	27.97	22.61
0.75	19.09	38.16	37.13
1	0	48.75	51.25

Supplementary Table 2. Peak area of polysulfanes in the supernatant of Cys-nFeS by LC-MS/MS (MRM).

Sulfides	Fe_3O_4[a]	$Cys_{0.5}$-nFeS [a]	$Cys_{1.0}$-nFeS [a]	Fe_3O_4 [b]	$Cys_{0.5}$-nFeS [b]	$Cys_{1.0}$-nFeS [b]	Fe_3O_4[c]	$Cys_{0.5}$-nFeS[c]	$Cys_{1.0}$-nFeS[c]
HSSH	0	29	15	0	429	467	0	906	474
HSSSH	0	64	100	0	344	368	0	908	1505
Cys-SSH	0	1	2	0	25	28	0	29	26
Cys-SSSH	0	36	27	0	50	113	0	102	205

Notes: Incubation time for Cys-nFeS to release polysulfanes in the supernatant. a: 0.5 h. b: 48 h. c: 96 h.

Supplementary Table 3. Parameters of kinetics assay for peroxidase-like activity with varied [H_2O_2].

Cysteine (g)	0	0.1	0.25	0.5	0.75	1.0
Vmax (nM s^{-1})	419.1	724.6	491.2	348.9	206.5	207.8
K_M (mM)	172.3	68.66	25.66	15.34	3.721	2.426
$Vmax/K_M$	2.43	10.55	19.14	22.74	55.5	85.66

Supplementary Table 4. **Parameters of kinetics assay for peroxidase-like activity with varied [TMB]**.

Cysteine (g)	0	0.1	0.25	0.5	0.75	1.0
Vmax (nM s^{-1})	9113	14124	24763	27857	28425	34896
K_M (μM)	1807	1863	3577	2793	2883	4470
$Vmax/K_M$	5.04	7.58	6.92	9.97	9.86	7.81

Supplementary Table 5. **Specific activity for peroxidase-like activity of Cys-nFeS**.

Materials	Specific activity (U mg^{-1})	s.d.
Fe_3O_4	0.061567	0.004632
$Cys_{0.1}$-nFeS	0.244333	0.007024
$Cys_{0.25}$-nFeS	0.278000	0.011533
$Cys_{0.5}$-nFeS	0.596333	0.000577
$Cys_{0.75}$-nFeS	0.371333	0.009074
$Cys_{1.0}$-nFeS	0.396667	0.003786
